# Unusual manifestations of adrenal insufficiency: A case report of hypopituitarism and Well's syndrome after apoplexy of a silent pituitary gonadotropic adenoma

**DOI:** 10.1097/MD.0000000000029274

**Published:** 2022-06-03

**Authors:** Chia-Chen Hsu, Hong-Da Lin, Chung-Yen Huang, Yi-Lun Chiang

**Affiliations:** a Division of Endocrinology, Shin-Kong Wu Ho-Su Memorial Hospital, Taipei, Taiwan; b Department of Medicine, Taipei-Veterans General Hospital, Taipei, Taiwan.

**Keywords:** adrenal insufficiency, case report, eosinophilia, pituitary apoplexy, Well's syndrome

## Abstract

**Rationale:**

Pituitary apoplexy occurs in about 8% of those with nonfunctioning pituitary adenoma. Subsequent hormone deficiency, especially corticotropic deficiency, is the most common finding. We describe the unusual manifestations of adrenal insufficiency that are usually overlooked in such cases, with the aim of raising awareness of this disease.

**Patient concerns:**

A 53-year-old male with a history of hyponatremia came to our hospital with intermittent fever and generalized pruritic skin rash. He also reported general weakness, abdominal pain, poor appetite, and severe retroorbital headache.

**Diagnoses:**

Laboratory data revealed hypereosinophilia, hypotonic hyponatremia, and hypopituitarism, including secondary adrenal insufficiency. Sellar magnetic resonance imaging revealed a pituitary macroadenoma, 2 cm in height, with mild displacement of the optic chiasm. Pathologic report and immunohistochemical stains of surgical specimen showed pituitary gonadotropic adenoma with apoplexy.

**Interventions:**

Transsphenoidal removal of the pituitary adenoma was performed. The patient received intravenous hydrocortisone then oral form cortisone acetate regularly.

**Outcomes:**

His symptoms and laboratory data recovered after the operation and medical treatment.

**Lessons:**

This case highlights that eosinophilia, pruritic skin rash and fever can be manifestations of adrenal insufficiency, and that they may initially be regarded as cellulitis.

## 1 Introduction

The pituitary gland lies in the sella turcica and it receives rich blood supply from many arteries. Pituitary tumors are mainly supplied by the inferior hypophyseal artery, and an enlarged tumor may compress the superior hypophyseal artery, which provides blood supply to most parts of the anterior pituitary gland, resulting in infarction or hemorrhage, called pituitary apoplexy. This can then result in hormone deficiency of the pituitary gland.^[1]^ Corticotropic deficiency is the most common hormone deficiency. Cortisol is associated with apoptosis and reduced production of eosinophils, and thus adrenal insufficiency can lead to eosinophilia.^[6]^ Eosinophilic cellulitis (Well's syndrome), presenting as an erythematous pruritic skin rash, is also a manifestation of adrenal insufficiency.^[7]^ Furthermore, fever is related to elevated C-reactive protein levels in patients with adrenal insufficiency.^[4]^ Hence, clinicians should always keep adrenal insufficiency in mind in patients with fever of unknown origin and a high C-reactive protein level, accompanied with pruritic skin rash which does not respond to antibiotic treatment.

Here, we present a case of pituitary adenoma with pituitary apoplexy and symptoms of subsequent hormone deficiency.

## 2 Case report

**Figure 1:**
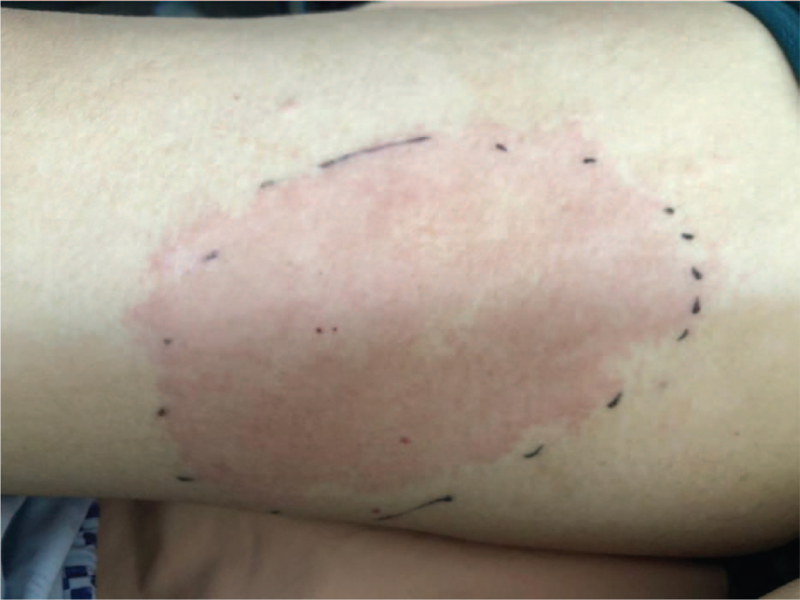
Pruritic, reddish, swelling skin plaque over arms.

**Figure 2:**
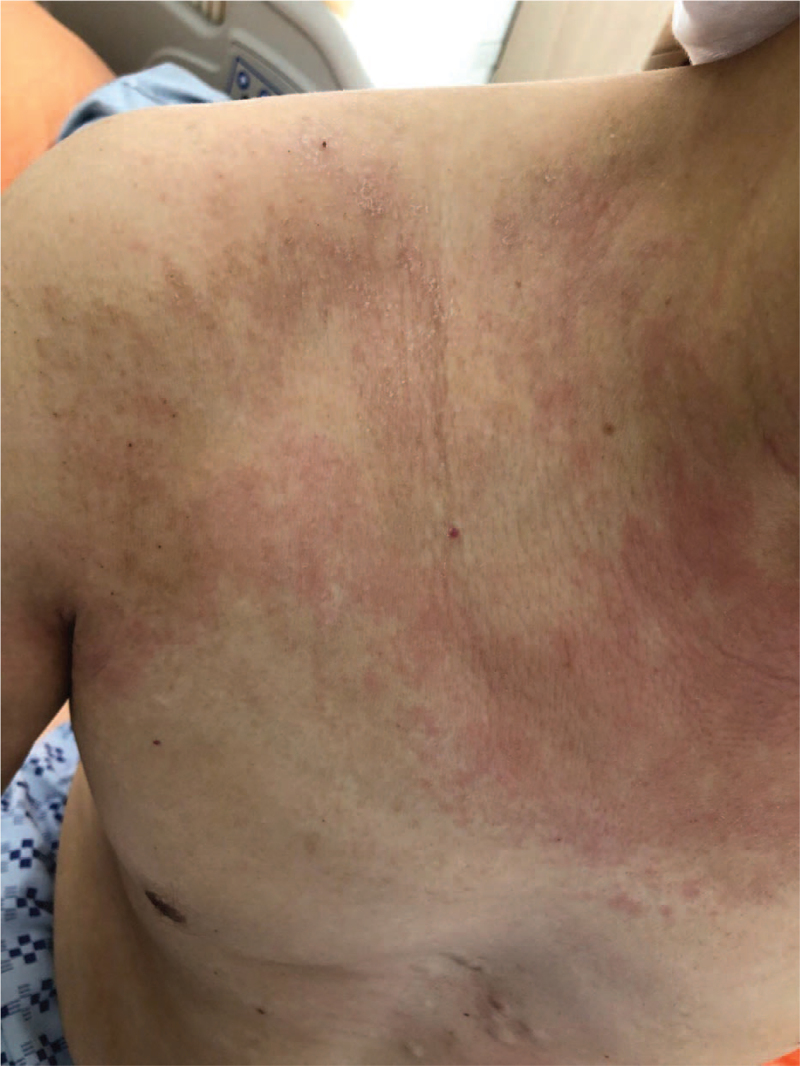
Pruritic, reddish, swelling skin plaque over the trunk.

A 53-year-old male patient was admitted to our hospital due to intermittent fever and pruritic skin rash over both arms and trunk for 4 days. He also complained of intermittent abdominal pain, general fatigue, and poor appetite for 6 months, with recent symptoms of a severe retroorbital headache. He had known allergies to trazodone which would cause a skin rash over his body, however he had not taken this medication before this presentation. He was discharged from another hospital 1 week before this presentation with the diagnoses of hyponatremia and urinary tract infection. The patient had no pertinent family history. A physical examination on admission revealed fever with a temperature of 38°C, heart rate of 96 beats/min, respiratory rate of 18 breaths/min, blood pressure of 116/68 mmHg, and peripheral capillary oxygen saturation (SpO_2_) of 98% under ambient air. Redness, swelling, and local heat were noted over both arms and trunk (Figs. 1 and 2). There was no pitting edema, and skin turgor was normal, compatible with euvolemic status. His muscle power was grade 5, and there were no neurological signs or visual disturbance.

**Figure 3:**
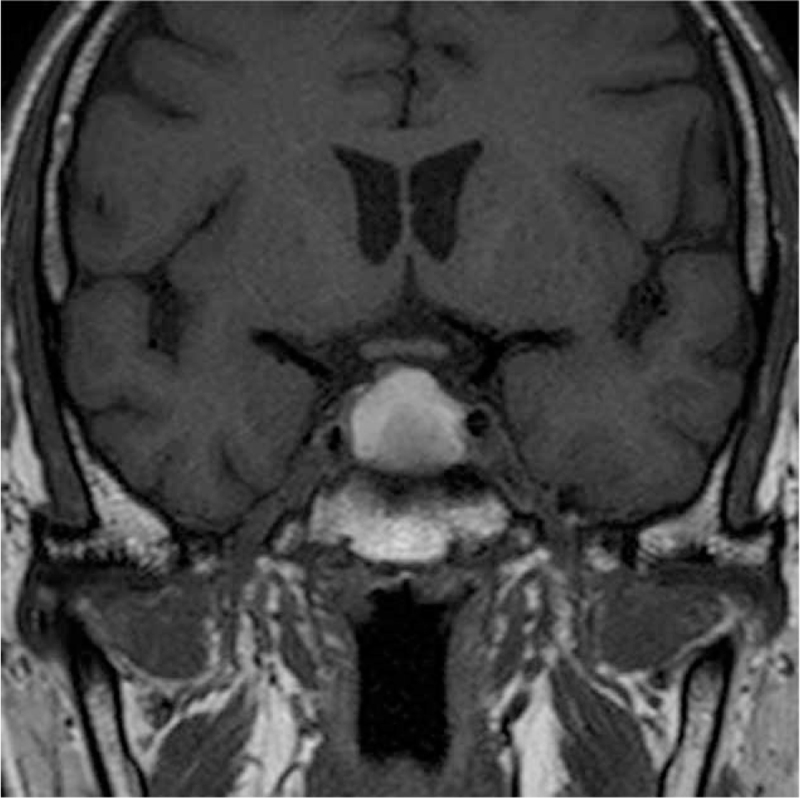
Coronal view of T1-weighted sellar MRI: a pituitary macroadenoma, 2 cm in height, with both intrasellar and suprasellar components. MRI = magnetic resonance imaging.

**Figure 4:**
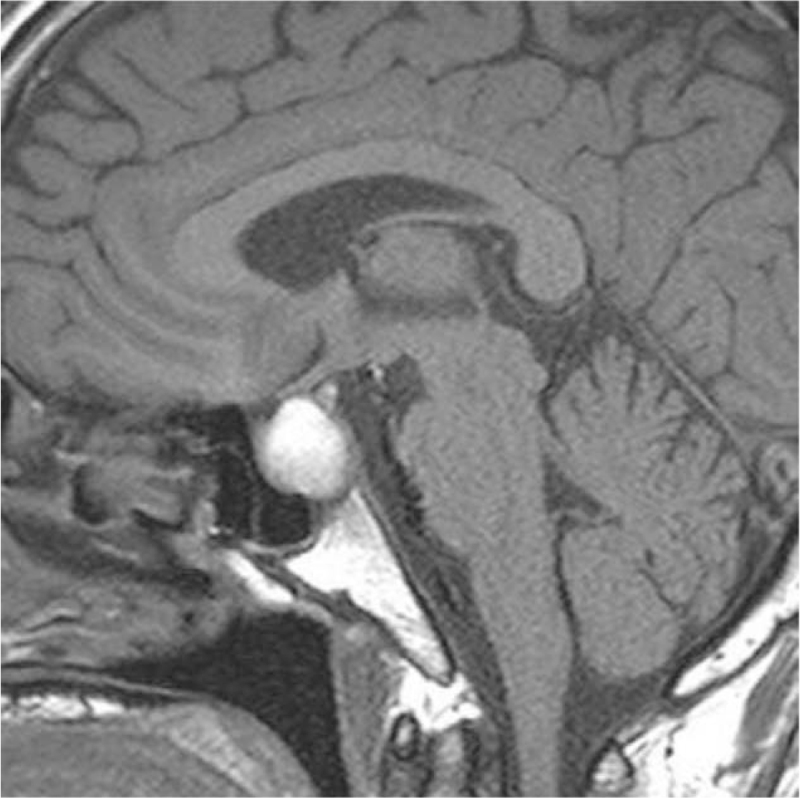
Sagittal view of T1-weighted sellar MRI: a pituitary macroadenoma with mild displacement of the optic chiasm. MRI = magnetic resonance imaging.

**Figure 5:**
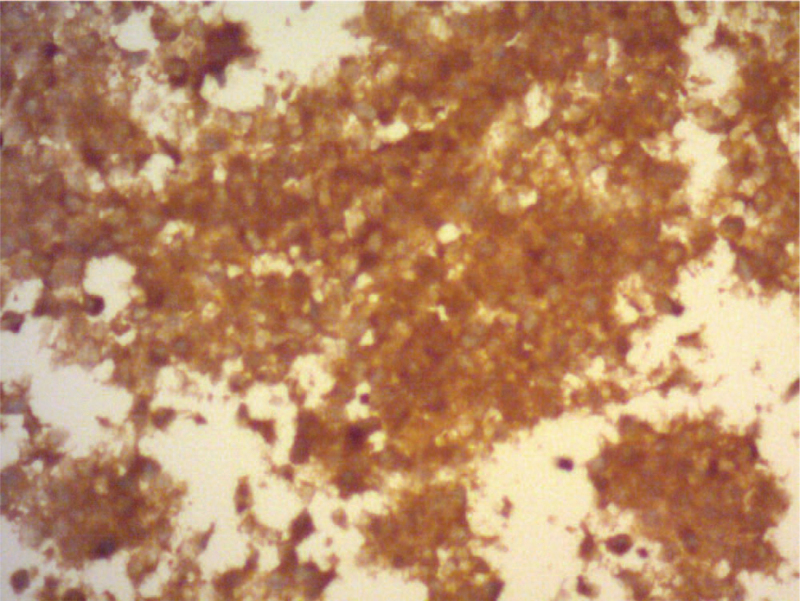
Immunohistochemical study shows that the tumor cells are reactive against FSH antibody. FSH = follicle-stimulating hormone.

On admission, his laboratory data (Table 1) revealed hypereosinophilia (white blood count: 8600/μL, eosinophil: 37%, eosinophil: 3182/μL), hypotonic hyponatremia (Na: 128 mmol/L, osmolarity: 265 mOsm/kg), normal potassium (K: 4.5 mmol/L), elevated C-reactive protein (CRP: 11.16 mg/dL), mildly elevated procalcitonin (0.2 ng/mL), and normal immunoglobulin E (14.8 IU/mL). Parasite ova-concentration collected from stool showed negative findings. Anti-neutrophil cytoplasmic antibodies, JAK2V617F, and tumor markers including carcinoembryonic antigen (0.9 ng/mL) and carbohydrate antigen 19-9 (9.5 U/mL) were checked for hypereosinophilia to rule out Churg-Strauss syndrome, myeloproliferative neoplasm, and malignancy-associated paraneoplastic hypereosinophilia, and all were normal. Urine biochemistry demonstrated Na: 88 mmol/L, osmolarity: 472 mOsm/kg. To rule out inappropriate antidiuretic hormone secretion as the cause of hyponatremia, we checked the cortisol and thyroid function, which showed low cortisol level (ante meridiem cortisol: 2.7 μg/dL) with normal adrenocorticotropic hormone (ACTH: 40.30 pg/mL), low free thyroxine 4 (0.40 ng/dL), low free T3 (1.56 pg/mL), elevated thyroid peroxidase antibody (803.5 IU/mL) and normal thyroid-stimulating hormone (TSH: 1.55 μIU/mL). We then checked other pituitary hormones to rule out hypopituitarism, and the results showed normal follicle-stimulating hormone (FSH: 5.2 mIU/mL), normal luteinizing hormone (LH: 3.79 mIU/mL), low testosterone (testosterone: 1.98 ng/mL), normal to low prolactin (prolactin: 3.7 ng/mL), normal human growth hormone (0.07 ng/mL), and low insulin-like growth factor-1 (39.70 ng/mL) for his age. Brain magnetic resonance imaging (MRI) (Figs. 3 and 4) revealed a pituitary macroadenoma, 2 cm in height, with mild displacement of the optic chiasm. An ophthalmologist found no visual field defects. Transsphenoidal removal of the pituitary adenoma was performed, and the neurosurgeon found a blood clot with necrotic tissue during surgery. The pathologist confirmed pituitary adenoma with apoplexy. Immunohistochemical studies showed that the tumor cells were reactive against FSH and synaptophysin antibodies (Fig. 5), weakly reactive against LH antibody, and not reactive against ACTH, prolactin, growth hormone and TSH antibodies. Pituitary gonadotropic adenoma with apoplexy was the final diagnosis.

**Table 1:** Serum, urine biochemistry and stool microscopic examination at admission.

Parameter (reference range)	First admission	After treatment
**Plasma**		
WBC (3.8–10.0 × 10^∗^3/μL)	8.6	6.4
Eosinophil (0%–5%)	37.0	4.6
Na^+^ (133–145 mmol/L)	128	142
K^+^ (3.3–5.1 mmol/L)	4.5	3.5
Osmolality (278–305 mOsm/kg)	265	287
CRP (0.00–1.00 mg/dL)	11.16	0.11
Procalcitonin (<0.12 ng/mL)	0.20	–
IgE (<160.0 IU/mL)	14.8	–
RA (0.0–14.0 IU/mL)	<10	–
ANA (Negative)	Negative	–
Anti-ENA screen (0.0–1.0 Ratio)	0.2	–
MPO ANCA (<5.00 IU/mL)	<0.20	–
PR3 ANCA (<3.00 IU/mL)	0.20	–
JAK2V617F (Undetected)	Undetected	–
CEA (0.0–5.0 ng/mL)	0.9	–
CA19–9 (0.8–35.0 U/mL)	9.5	–
IgG (700–1600 mg/dL)	984	–
IgG4 (3.0–201.0 mg/dL)	53.1	–
TSH (0.35–4.94 μIU/mL)	1.55	1.71
T4, Free (0.7–1.48 ng/dL)	0.40	0.79
T3, Free (1.88–3.18 pg/mL)	1.56	2.35
Anti-TPO (0.0–5.6 IU/mL)	803.5	–
Cortisol (6.7–22.6 μg/dL)	2.7	4.1
ACTH (5.0–77.0 pg/mL)	40.30	33.70
FSH (1.27–19.26 mIU/mL)^∗^	5.20	5.99
LH (1.24–8.62 mIU/mL)^∗^	3.79	2.63
Testosterone (1.750–7.810 ng/mL)	1.98	4.46
Prolactin (2.64–13.13 ng/mL)^∗^	3.70	6.05
IGF-1 (53-year-old: 64–218.0 ng/mL)	39.70	72.49
hGH (0.0–5.0 ng/mL)^∗^	0.07	0.27

Parameter (reference range)	First admission
**Spot urine**	
Na (mmol/L)	88
Osmolarity (mOsm/kg)	472
**Stool**	
Parasite Ova-Concentration (Not found)	Not found

∗ Indicates reference range for the men. ANA = an antinuclear antibody, ANCA = anti-neutrophil cytoplasmic antibodies, Anti-ENA screen = anti-extractable nuclear antigen antibody, Anti-TPO = anti*-*thyroid peroxidase Ab, CEA = carcinoembryonic antigen, hGH = human growth hormone, IgE = immunoglobulin G, IGF = insulin-like growth factor, RA = rheumatoid factor, T4 = thyroxine 4.

On admission, he was given oxacillin 500 mg every 6 hours for suspected cellulitis, however his pruritic skin rash did not subside. Before the operation, intravenous hydrocortisone 100 mg was administered every 8 hours, as well as the oral form of levothyroxine 50 mcg per day before meals. After the operation, cortisone acetate 25 mg in the morning/12.5 mg in the afternoon per day, and levothyroxine 50 mcg per day before meals were administered. The pruritic erythematous plaques subsided over both arms and trunk, and his eosinophil count and sodium level returned to normal.

## 3 Discussion

Pituitary apoplexy is induced by ischemia or hemorrhage of the pituitary gland. The most common cause is pituitary adenoma, which grows in the sella turcica and can compress the vessels that supply the pituitary gland, leading to ischemia, necrosis and hemorrhage of the pituitary gland.^[1]^ Pituitary apoplexy occurs in 2% to 12% of patients with all types of pituitary adenoma, and in about 8% of those with nonfunctioning pituitary adenoma. The peak incidence is during the fifth or sixth decade of life, with a male to female ratio ranging from 1.1 to 2.3:1.^[2]^ Even in a normal pituitary gland, pituitary apoplexy can be seen. Infarction of the pituitary gland can occur in pregnant women who lose a life-threatening amount of blood during delivery due to Sheehan's syndrome. The precipitating factors include hypertension, coagulopathy, dynamic testing of the pituitary gland, radiation therapy, surgery, trauma, and infection.^[1]^

The severe, sudden onset of headache behind the eyes, described as “a thunderclap in a clear sky” has been reported in more than 80% of patients. Visual disturbance, nausea, vomiting and symptoms resulting from hypopituitarism may also occur. Pituitary adenoma may cause hypopituitarism due to the loss of blood supply to the anterior pituitary gland, or increased intrasellar pressure on the pituitary stalk affecting the hypothalamus and/or pituitary gland to release hormones. This can then lead to isolated or greater hormone deficiency, and corticotropic deficiency is the most common finding, seen in 50% to 80% of cases.^[2]^ Prolactin level is usually elevated in patients with pituitary macroadenoma. This may be because the macroadenoma is a prolactinoma, which produces much more prolactin, or if the macroadenoma is not a prolactinoma, then it may limit the transmission of dopamine via the pituitary stalk, thereby diminishing the inhibitory effect. A normal to low prolactin level is not commonly seen in patients with pituitary macroadenoma. One possible reason is that high intrasellar pressure prevents the release of hormones, suggesting that these patients have severe pituitary apoplexy.^[1]^ Another possible reason is the high dose hook effect, which occurs when the serum specimen contains too much prolactin (such as pituitary macroprolactinoma). Prolactin level is calculated using the double antibody sandwich technique by producing a heterotrimeric complex (capture antibody-prolactin-reporter antibody). However, in cases of prolactinoma, there are insufficient antibodies in the testing agent to bind to both ends of prolactin, therefore leading to underestimation of the prolactin level.^[1,3]^ Our case was not a prolactinoma, and thus the normal to low prolactin was more likely due to severe pituitary apoplexy. ACTH deficiency is usually present with pituitary apoplexy. Some patients with adrenal insufficiency also have fever. Fever can be associated with infection, a factor which can induce pituitary apoplexy, and thus infection surveys should be performed simultaneously. In addition, a study that investigated the association between fever and CRP level in patients with adrenal insufficiency found a higher CRP level in febrile patients than in nonfebrile patients. This may be due to cytokine release induced by adrenal insufficiency. Some fever-producing substances promote the production of endogenous pyrogen and prostaglandin E_2_, causing fever in patients with adrenal insufficiency. In addition, CRP is produced in the liver due to stimulation by the cytokines interleukin-6 and interleukin-1β.^[4]^ Therefore, if a febrile patient with increased CRP is diagnosed with fever of unknown origin, the differential diagnosis should include adrenal insufficiency. The administration of steroids that reduce fever may be helpful to distinguish adrenal insufficiency from infection in febrile patients diagnosed with adrenal insufficiency, and it can also reduce the unnecessary use of antibiotics.^[4]^

Another feature of adrenal insufficiency is eosinophilia. The definition of eosinophilia is an absolute eosinophil count > 500/mm^3^ in peripheral blood, and hypereosinophilia is defined as a persistent absolute eosinophil count > 1500/mm^3^.^[5]^ The factors affecting serum eosinophil count include the production rate in bone marrow, and the expression time in peripheral blood. A previous case report of a patient with adrenal insufficiency and eosinophilia receiving steroid treatment reported a 50% decrease in eosinophils in bone marrow, and a 66% decrease in marrow eosinophils in deoxyribonucleic acid synthesis (S phase). Steroids stimulate the migration of eosinophils from blood vessels to tissues, and also induce the apoptosis of eosinophils. Furthermore, steroids reduce the production rate in bone marrow, which may take 2 days to influence the serum eosinophil level. Taken together, these findings suggest that cortisol deficiency may result in eosinophilia.^[6]^

Well's syndrome (eosinophilic cellulitis) is an inflammatory skin disease with edema and eosinophil infiltration in the dermis seen on skin biopsy.^[7,9]^ Approximately 50% of patients with Well's syndrome have blood eosinophilia. The levels of eosinophils may fluctuate during the course of the disease, and become normal after clinical resolution.^[8]^ The etiology of Well's syndrome is still unclear. Some authors have proposed that the triggering factors of this disease may include insect bites, bacterial or viral infection, drug eruption or thimerosal-containing vaccines. Other authors have suggested that this disease may be associated with lymphoproliferative malignancies and carcinoma.^[9]^ One of the underlying causes is adrenal insufficiency.^[7]^ The skin manifestations can be separated into 2 stages. First, it presents as localized or diffuse erythematous plaques, with a pruritic or burning sensation. Mild tenderness and subsequent edematous change then occur. Moreover, papules, nodules, blisters or bullae may be seen. The second stage lasts 2 to 8 weeks with the progression of skin lesions, leading to hyperpigmentation and morphea-like residual skin atrophy. Its clinical manifestations are similar to bacterial cellulitis; however, it does not respond to antibiotics. The most effective treatment for Well's syndrome is oral steroids, with a treatment success rate of 91.7%. Oral prednisolone 2 mg/kg per day can be used for 1 week, and then gradually tapered for 2 to 3 weeks. Other choices such as topical steroids and antihistamines achieve lower success rates of 50% and 25%, respectively.^[9]^ Because it is usually misdiagnosed as infectious cellulitis, Well's syndrome should be 1 of the differential diagnosis for cellulitis with atypical features which cannot be treated by antibiotics.^[7,9]^ Although our patient did not receive a skin biopsy, his pruritic and reddish skin rash did subside after steroid use, suggesting that it was Well's syndrome.

In addition, our patient was positive for antithyroid peroxidase antibodies, and therefore, we suspect that the patient had autoimmune thyroid disease before. Another cause is adrenal insufficiency. A high serum cortisol level is related to immunosuppression, and thus cortisol deficiency may lead to accelerated autoimmunity, such as elevated antithyroid peroxidase Ab.^[10]^ Schmidt syndrome is a combination of primary adrenal insufficiency (Addison disease) and hypothyroidism and/or type 1 diabetes mellitus.^[11]^ However, our case did not have Schmidt syndrome.

Nonfunctioning pituitary adenoma is defined as pituitary adenoma without excess hormone production. Immunohistochemistry staining can be used for many types of tropic hormones, of which the most common type is silent gonadotropic adenoma, which accounts for 80% of resected nonfunctioning pituitary adenomas, followed by corticotropic adenoma, pituitary transcription factor-1 (GH/prolactin/TSH) lineage, then null cell adenoma. Null cell adenoma is defined as an adenoma with negative immunohistochemistry staining results for hormones and transcription factors. The pituitary tumor in our case was confirmed to be a gonadotropic adenoma as it only showed a positive response to FSH. In some equivocal cases, steroidogenic factor 1 can be used to differentiate a silent gonadotropic adenoma from other types, and many LH/FSH immunonegative adenomas are actually found to be silent gonadotroph adenomas according to positive steroidogenic factor 1 on immunostaining. Estrogen receptor-α is a prognostic factor for silent gonadotroph adenoma in males, and the absence of estrogen receptor-α with a young age is associated with a good prognosis. Nonfunctioning pituitary adenomas are usually incidentally found on imaging studies or autopsy. Therefore, some cases are found when the tumor grows larger, leading to a mass effect, as in our case. Indications for surgery include interference of the optic chiasm or rapid enlargement of the tumor. For asymptomatic microadenomas, brain MRI should be performed every year in the first 3 years, with gradually prolonged follow-up times, to detect tumor growth. For tumors < 5 mm in size, no further imaging studies are needed. For asymptomatic macroadenomas, the visual field should be examined, and brain MRI should be performed every 6 months, then annually for 3 years.^[12]^

The limitation of this case report is the lack of similar reports for citation, because such presentation of adrenal insufficiency is not commonly seen.

## 4 Conclusion

Pituitary apoplexy usually occurs in patients with pituitary adenoma, and is defined as ischemia or hemorrhage of the pituitary gland. Hypopituitarism followed by decreased blood supply to the anterior pituitary gland may develop, and corticotropic deficiency is the most common finding. Unusual manifestations of adrenal insufficiency include eosinophilia and eosinophilic cellulitis (Well's syndrome), which is characterized by pruritic, erythematous and edematous skin plaques, and may suggest bacterial cellulitis. Antibiotic treatment is not always efficient. This case report highlights that if patients are diagnosed with fever of unknown origin, especially when they have atypical findings as described above, adrenal insufficiency should be considered to avoid the inappropriate use of antibiotics.

## Author contributions

Chia-Chen Hsu MD analyzed and interpreted the imaging findings and laboratory data, reviewed the literature and contributed to drafting the manuscript; Yi-Lun Chiang MD, Hong-Da Lin MD, PhD, Chung-Yen Huang MD made critical revisions related to important intellectual content of the manuscript; all authors issued final approval for the version to be submitted.

**Conceptualization:** Chia-Chen Hsu, Hong-Da Lin

**Data curation:** Chia-Chen Hsu

**Formal analysis:** Chia-Chen Hsu

**Resources:** Chia-Chen Hsu

**Software:** Chia-Chen Hsu

**Supervision:** Chung-Yen Huang, Hong-Da Lin, Yi Lun Chiang

**Validation:** Hong-Da Lin, Yi Lun Chiang

**Visualization:** Chung-Yen Huang, Hong-Da Lin, Yi Lun Chiang

**Writing – original draft:** Chia-Chen Hsu

**Writing – review & editing:** Chia-Chen Hsu
